# Patient Versus Clinician Reported Symptoms Agreement in Advanced Metastatic Bladder Cancer Patients

**DOI:** 10.1002/cam4.70896

**Published:** 2025-04-21

**Authors:** Soufyan Annakib, Emma Di Méglio, Yona Dibert‐Bekoy, Thierry Chevallier, Guilhem Roubaud, Pierre Fournel, Aline Guillot, Delphine Borchiellini, Damien Pouessel, Elouen Boughalem, Remy Delva, Philippe Barthelemy, Stéphane Oudard, Constance Thibault, Diego Tosi, Nadine Houédé, Frédéric Fiteni

**Affiliations:** ^1^ Medical Oncology Department, CHU Nîmes, Univ Montpellier Nîmes France; ^2^ Department of Biostatistics, Epidemiology, Public Health and Innovation in Methodology (BESPIM), CHU Nîmes, Univ Montpellier Nîmes France; ^3^ University of Montpellier, UMR INSERM IDESP—Desbrest Institute of Epidemiology and Public Montpellier France; ^4^ Medical Oncology Department Institut Bergonié Bordeaux France; ^5^ Medical Oncology Department Institut de cancérologie Lucien Neuwirth Saint‐Etienne France; ^6^ Medical Oncology Department Centre Antoine Lacassagne, Université Côte D'azur Nice France; ^7^ Medical Oncology Department Institut Universitaire du Cancer de Toulouse Oncopole, Oncopole Claudius Regaud Toulouse France; ^8^ Medical Oncology Department Institut de Cancérologie de L'Ouest Site Paul Papin Angers France; ^9^ Medical Oncology Department Institut de Cancérologie Strasbourg Europe Strasbourg France; ^10^ Medical Oncology Department Hôpital Européen Georges Pompidou, université Paris Cité Paris France; ^11^ Medical Oncology Department Institut régional du Cancer de Montpellier Montpellier France; ^12^ Institut de Recherche en Cancérologie de Montpellier (IRCM), Institut régional du Cancer Montpellier, INSERM U1194 Université de Montpellier Montpellier France

**Keywords:** adverse drug events, bladder cancer, immune checkpoint inhibitors, patient reported outcome, pembrolizumab, PRO‐CTCAE

## Abstract

**Background:**

Immune checkpoint inhibitors (ICIs) improved survival in patients with locally advanced or metastatic urothelial carcinoma (la/mUC). Patient‐reported symptoms in this context were poorly studied. The study aimed to compare symptom severity between patients and clinicians.

**Methodology:**

The secondary analysis of the AMI clinical trial comparing changes in the gut microbiota in patients with la/mUC treated with pembrolizumab was conducted in nine French centers. Secondary endpoints were expected in this prospective study. Patient‐Reported Outcome‐Common Terminology Criteria for Adverse Events (PRO‐CTCAE) and CTCAE were assessed respectively by patients and clinicians before pembrolizumab initiation, and at each treatment visit until treatment cycle 12. Agreement in severity between clinicians and patients for grade ≥ 3 symptoms was calculated with Cohen's kappa coefficient. The toxicity index was generated for CTCAE and PRO‐CTCAE to assess discordance in a longitudinal manner. The Wilcoxon test was used to compare clinicians' and patients' toxicity index and symptom severity frequencies.

**Results:**

Thirty‐nine patients were included (M/F sex ratio: 2.5) from December 2020 to March 2022. PRO‐CTCAE baseline completion rate was 77.5%. Cohen's kappa coefficient ranged from −0.017 (95% confidence interval (CI), [−0.039, 0.005]) for numbness/tingling to 0.161 (95% CI, [0.045, 0.276]) for fatigue. The patient self‐rated symptom toxicity index was > 2 for all symptoms compared to ≤ 0.62 (fatigue) when assessed by clinicians in longitudinal reporting of symptom frequency and severity with a *p* value < 0.001. The three most commonly reported symptoms by patients and clinicians, respectively, were: Fatigue 53.3% versus 23.4%, generalized pain 42.4% versus 16.5%, and insomnia 41.1% versus 9.5%. Symptom frequency reports between clinicians and patients were statistically different (*p* < 0.009).

**Conclusions:**

Symptom severity assessment showed discordance between patients and physicians. Clinicians reported fewer symptoms and graded them less severely than patients. PROs should be used to accurately reflect patient experience.

**Trial Registration:**

ClinicalTrials.gov identifier: NCT04566029

## 1 Background

Pembrolizumab is an anti‐Programmed cell Death protein‐1 (anti‐PD1) approved in second‐line treatment for locally advanced or metastatic urothelial carcinoma (la/mUC) [1]. As with other immune checkpoint inhibitors (ICI), the side effects of pembrolizumab are referred to as immune‐related side effects. They can quickly worsen and lead to more medical complications [2, 3]. AEs are reported using the Common Terminology Criteria for Adverse Events (CTCAE) divided into three major categories (laboratory results, technical measures and subjective symptoms) [4]. Although the CTCAE is widely used in clinical trials for AEs assessment, it does not assess the patients' perception of frequency and severity of symptoms. Patient reported outcomes (PRO) are usually evaluated using health‐related quality of life (HRQoL) questionnaires. These tools generally include questions related to symptoms. Interestingly, studies comparing symptom or AEs measurement agreement between clinicians (using CTCAE instrument) and patients (HRQoL instruments) have shown that clinicians underestimate the frequency and severity of subjective symptoms [5, 6]. Indeed, patients generally reported symptoms earlier and more frequently than clinicians [5]. However, poor data on this disparity are available in la/mUC patients treated with ICIs [7].

The Patient Reported Outcome‐CTCAE (PRO‐CTCAE) is a complementary tool to CTCAE measuring patient self‐report of symptoms and AEs. This is a standardized tool that has demonstrated validity, reliability, and responsiveness in a large, heterogeneous sample of patients undergoing different cancer treatments [8, 9]. It is an easy tool to understand and is relevant to prospective comparative effectiveness research. It is also validated for multicenter studies with low administrative requirements [10, 11, 12]. The definition of PRO‐CTCAE item selection is not well established, and the choice of symptoms is left to the investigator's discretion. Symptoms to be included could be selected according to a literature review of frequent symptoms, according to medical team (nurse + clinician) experience, by patients already under treatment, or based on health authorities' registered drug characteristics consultation [13, 14, 15, 16].

CTCAE and PRO‐CTCAE reports are usually based on the frequency of the highest‐grade symptoms, without information about longitudinal and cumulative toxicity over time [17]. Thus, toxicity scoring algorithms, as the toxicity index has been developed to account for these issues, generating a single score [18, 19, 20, 21, 22, 23, 24, 25]. The toxicity index is a normalized score for PRO‐CTCAE and CTCAE and provides a direct comparison between patients' and clinicians' reports, considering the evolution of symptom severity during follow‐up.

Our study aimed to compare agreement between clinicians and patients for symptom severity assessed using the PRO‐CTCAE and CTCAE instruments in la/mUC patients treated with pembrolizumab. We hypothesized that clinicians would systematically underestimate patients' symptom severity and frequency.

## 2 Methods

### 2.1 Study Design and Patients

The AMI clinical trial (www.clinicaltrials.gov, registered on 28 September 2020, NCT04566029) is a prospective study with nine recruiting French oncological centers. The study was conducted in accordance with the Declaration of Helsinki and approved by the French ethical committee (*Comité de protection des personnes*, #2020/56). All patients provided written informed consent to participate. The primary outcome of this study was gut microbiota change over treatment. These results will be presented in another publication. Here, we present one of the key secondary outcomes: to compare the concordance between patients and clinicians for symptoms severity report using PRO‐CTCAE and CTCAE instruments.

Eligible patients were patients with histologically proven la/mUC with an indication for pembrolizumab. Patients previously treated with ICI were excluded. Patients completed the paper PRO instrument before starting treatment and at each treatment cycle (every 3 weeks) for the first six pembrolizumab cycles and at the twelfth cycle. The PRO instruments included the PRO‐CTCAE, European Organization for Research and Treatment of Cancer Quality of Life Questionnaire Core 30 (EORTC QLQ‐C30) and EuroQoL 5‐Dimensions 5‐Level (EQ‐5D‐5L). HRQoL results will be reported in a separate article. The planned maximum follow‐up period was 36 weeks. Clinicians evaluated the presence and the severity of the same symptom using a CTCAE form with predefined symptoms blinded to the patient‐reported scores.

### 2.2 Adverse Events Assessment

The PRO‐CTCAE includes 124 items representing 78 symptomatic toxicities drawn from the CTCAE based on a 7‐day recall period and is completed by patients [26]. Each symptom is graded according to the frequency, severity, and/or activity interference. Among the 78 symptoms of the PRO‐CTCAE, we selected 20 symptoms (36 items) based on the most frequently reported pembrolizumab AEs identified in the KEYNOTE 045 phase III clinical trial [1]. Symptom frequency and severity were collected at baseline and at each scheduled visit, and patients graded severity from 1 (mild severity) to 4 (very severe). The same symptoms in the CTCAE version 4.03 response grid were used by clinicians. Detailed information about how to grade severity was available for each symptom. Clinicians graded severity from 1 (mild‐minor symptom) to 4 (life‐threatening consequences) and 5 (death). The absence of a symptom severity report by clinicians was considered as absence of the symptom. Clinicians and patients completed the instruments in a blinded manner.

### 2.3 Statistics

The completion rate was calculated as the proportion of patients or clinicians who completed the questionnaire among the total enrolled patients. The compliance rate was defined as the proportion of patients or clinicians who completed questionnaires at each visit among those expected to complete the questionnaire at each visit (excluding patients who discontinued the study). For the CTCAE questionnaire, non‐answered items were considered as absence of symptom if the questionnaire was completed, or missing information if not completed. Missing values were not replaced.

Symptom frequencies by grade were generated for each scheduled visit for all symptoms. Toxicity index was generated for each patient for CTCAE and PRO‐CTCAE according to previously reported statistical method [23, 25]:

$$\sum_{i\leq m}\frac{x_{i}}{\pi_{j<i}\left(1+x_{j}\right)}$$

*m*: number of AEs for a given patient. *x*_*i*_: *x*_*i=*1_ is the largest AE grade, *x*_*i=*2_ is the second largest AE grade, and so on, up to the smallest AE grade, *x*_*i=m*_.

**FIGURE 1:**
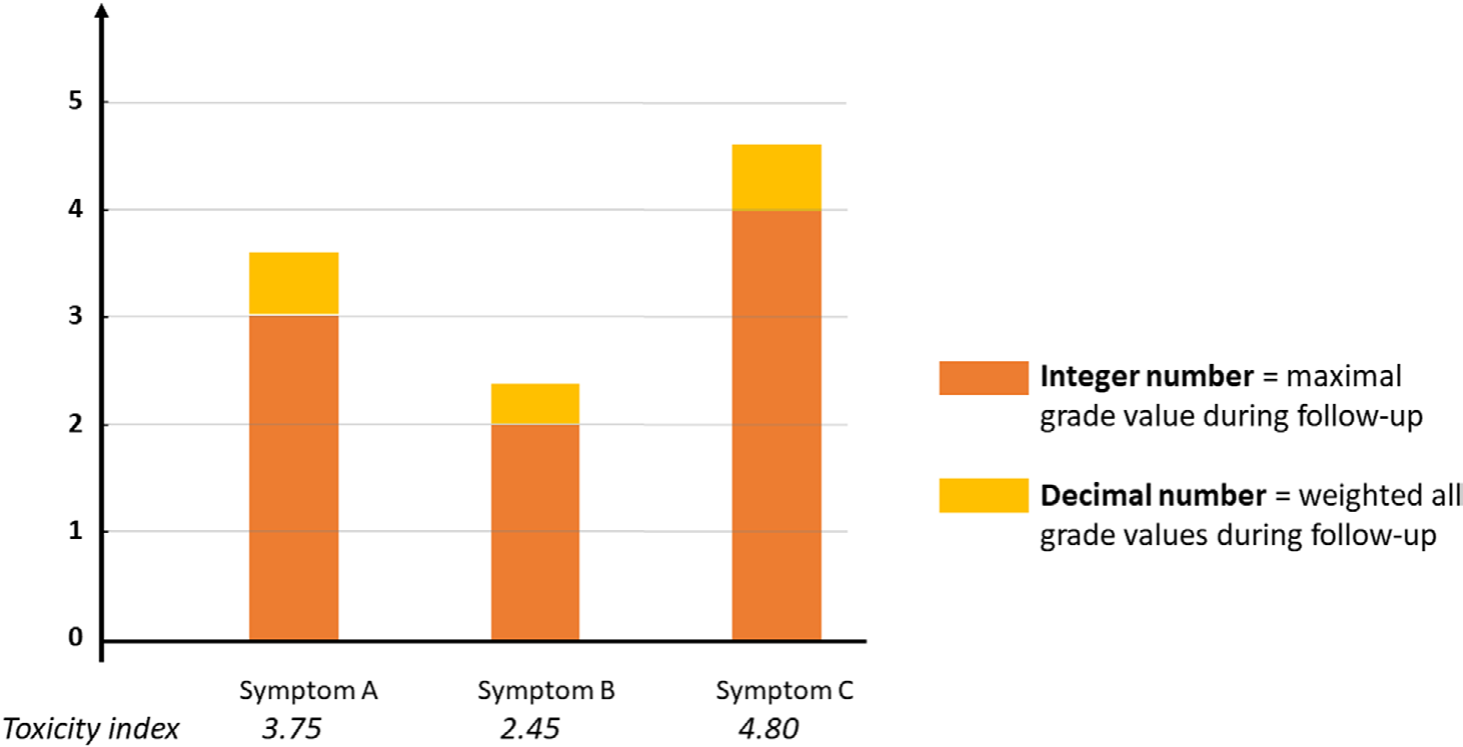
Toxicity index interpretation. Each bar corresponds to the toxicity index value ranged from “0” to “4.999”.

The toxicity index integer value corresponded to the highest symptom grade experienced during follow‐up; the decimal value corresponded to the weighted addition of all other symptom grades experienced by the patients (Figure 1).

To test the patient‐clinician agreement, symptom severity was rated as concordant by grade matching until grade 4. Three different statistical methods were used to compare accordance between clinicians and patients:

– A Cohen's kappa model was used for symptom severity comparative analysis. To compare the accordance between clinicians and patients for one symptom graded ≥ 3, patients and clinicians both needed to report at least one event grade ≥ 3. Concordance was classed as strong (*ĸ* = 0.80–1.00), good (*ĸ* = 0.60–0.79), moderate (*ĸ* = 0.40–0.59), fair (*ĸ* = 0.20–0.39), poor (*ĸ* = 0.00–0.19), and bad (*ĸ* < 0) [27, 28].
– Next, we compared the toxicity index between clinicians and patients at the individual level using the Wilcoxon test for non‐matched data.
– We used a Wilcoxon test to compare differences in symptom severity frequency report.

All statistical analyses were done using R software version 4.1.1 (R Development Core Team (200918), R Foundation for Statistical Computing, Vienna, Austria). The alpha risk was defined at 5% for all the statistical tests.

## 3 Results

### 3.1 Study Participants

From December 2020 to March 2022, 40 subjects were enrolled, with one withdrawing consent before treatment initiation, thus 39 (97.5%) subjects were analyzed. Data collection was finalized on March 2023 for this analysis. Baseline study participants characteristics are summarized in Table 1. Of the 39 subjects, 28 (71.8%) were male and 11 (28.2%) were female. Median age was 72.2 [range, 51–85]. Eastern Cooperative Oncology Group Performance Status (ECOG PS) was ≥ 2 in 8 (20%) subjects. Locally advanced presentation form (local disease relapse or pelvic nodes extension) was seen in 12 (30.8%) subjects. Main histological subtype was pure urothelial carcinoma (46.2%), with the presence of a non‐urothelial histological subtype in 6 (15.4%) subjects. Before pembrolizumab treatment, 14 (35.9%) and 23 (59%) subjects received carboplatin and cisplatin‐based regimens, respectively. Twelve (30.8%) and 6 (15.4%) subjects had, respectively, a history of neoadjuvant or adjuvant treatment.

**TABLE 1:** Study participants baseline characteristics.

	Patients (*n*)	Percentage
Sex		
Male	28	71.8
Female	11	28.2
Age (years old)		
Median	72.2	0
Range	[51–85]	0
ECOG PS		
0;1	32	80
≥ 2	8	20
Primary tumor site		
Bladder	31	79.5
Upper tract	8	20.5
Histologic testing		
Pure urothelial carcinoma	18	46.2
Predominance of urothelial‐cell features	11	28.2
Presence of non‐urothelial form (a)	6	15.4
Missing	4	10.3
Disease extension		
Locally advanced (b)	12	30.8
Liver metastasis	2	5.1
Lung metastasis	2	5.1
Extrapelvic lymph nodes	8	20.5
Other sites	15	38.5
Hemoglobin concentration (g/dL)		
< 10	11	28.2
≥ 10	28	71.8
Neutrophil‐to‐lymphocyte ratio		
< 5	20	51.3
≥ 5	19	48.7
History of perioperative chemotherapy		
Neoadjuvant treatment	12	30.8
Adjuvant treatment	6	15.4
None	19	48.7
Missing	2	5.1
History of bladder surgical reconstruction		
Bricker	10	25.6
Conservative treatment	7	18.0
Missing	22	56.4
First‐line systemic treatment		
Cisplatin‐based chemotherapy	23	59.0
Carboplatin‐based chemotherapy	14	35.9
Missing	2	5.1

*Note:* (a) Histological non‐urothelial forms: micro‐papillary, microcystic, trophoblastic differentiated, nested, plasmacytoid, sarcomatoïd, rhabdoid, lymphoepitheliomatoïd, clear cell, large cell, undifferentiated urothelial carcinoma. (b) Local relapse or pelvic nodes positive.

Abbreviations: ECOG PS: eastern cooperative oncology group performance status; LDH: lactate dehydrogenase.

### 3.2 Patient‐Reported Outcome Questionnaire Completion and Compliance

Over the total study, 231 questionnaires were completed. The PRO‐CTCAE baseline completion and compliance rates were 77.5%. The completion rate increased over time up to 85.2% at visit 4 and then decreased to 69.2% at visit 12. Detailed completion and compliance rates at each timepoint are reported in Table 2.

**TABLE 2:** PRO‐CTCAE completion and compliance rates at baseline and at scheduled visits.

Visit	Number of subjects per visit	Completion rate per visit (%)	Compliance rate per visit (*n* = 40) (%)
Baseline	40	77.5	77.5
Visit 1	39	64.1	62.5
Visit 2	37	83.8	77.5
Visit 3	33	66.7	55.0
Visit 4	27	85.2	57.5
Visit 5	23	56.5	32.5
Visit 6	24	50.0	30.0
Visit 12	13	69.2	22.5

Abbreviation: PRO‐CTCAE: patients reported outcomes‐common terminology criteria for adverse events.

### 3.3 Agreement for Symptom Reporting

**FIGURE 2:**
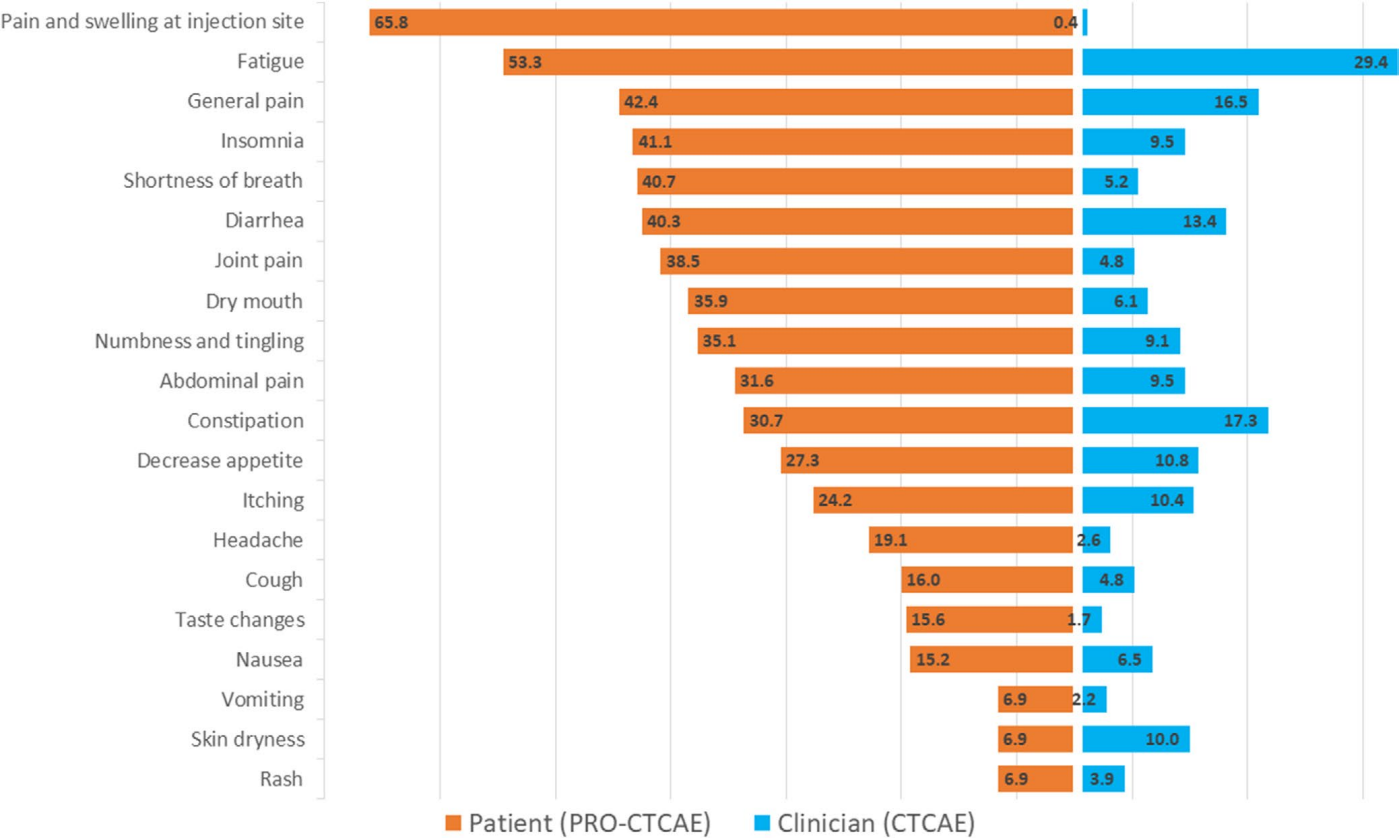
Symptom frequency using CTCAE and PRO‐CTCAE instruments assessment across all study follow‐up. Results are reported in percentages for each symptom. CTCAE: common terminology criteria for adverse events; PRO‐CTCAE: patient reported outcomes‐common terminology criteria for adverse events.

Across all visits, all symptoms were reported by at least one patient and one clinician (Figure 2). The symptoms most frequently reported by patients vs. clinicians were respectively: Pain and swelling at the injection site 65.8% vs. 0.4%, fatigue 53.3% vs. 23.4%, general pain 42.4% vs. 16.5%, insomnia 41.1% vs. 9.5%, shortness of breath 40.7% vs. 5.2%, diarrhea 40.3% vs. 13.4%, joint pain 38.5% vs. 4.8%, dry mouth 35.9% vs. 6.1%, numbness and tingling 35.1% vs. 9.1%, abdominal pain 31.6% vs. 9.5%. All symptom frequencies are shown in Figure 2.

When comparing the grade ≥ 3 symptom frequencies, patients and clinicians reported respectively (Table 3 and Table S1): 23.4% vs. 2.6% for fatigue (*p* < 0.001), 10.8% vs. 4.3% for general pain (*p* < 0.001), 10.0% vs. 1.3% for decreased appetite (*p* < 0.001), 10.8% vs. 0.9% for abdominal pain (*p* < 0.001), 12.4% vs. 0.9% for numbness and tingling (*p* < 0.001), 13.9% vs. 0.4% for dry mouth (*p* < 0.001), 4.3% vs. 0.4% for nausea (*p* = 0.008), 19.9% vs. 0.4% for constipation (*p* < 0.001), 6.9% vs. 0.4% for skin dryness (*p* < 0.001). All other symptoms were graded as grade ≥ 3 by at least one patient, but not by clinicians (0%) (*p* < 0.002).

**TABLE 3:** Grade severity of each symptom according to patient and clinician assessment across follow‐up.

Symptom	PRO‐CTCAE		CTCAE		KEYNOTE 045 trial (d)	
	All grade (%)	Grade ≥ 3 (%)	All grade (%)	Grade ≥ 3 (%)	All grade (%)	Grade ≥ 3 (%)
Dry mouth (a)	35.9	13.9	6.1	0.4	2.3	0.4
Taste changes	15.6	6.9	1.7	0	2.6	0
Decreased appetite	27.3	10.0	10.8	1.3	21.1	3.8
Nausea	15.2	4.3	6.5	0.4	20.7	0
Vomiting	6.9	2.6	2.2	0	14.7	1.5
Constipation	30.7	19.9	17.3	0.4	18.8	1.1
Diarrhea (b)	40.3	—	13.4	0	16.2	1.5
Abdominal pain	31.6	10.8	9.5	0.9	12.8	1.1
Shortness of breath	40.7	14.3	5.2	0	12.4	1.9
Cough	16.0	6.1	4.8	0	14.3	0.4
Rash (b)	6.9	—	3.9	0	10.9	0.4
Skin dryness	33.8	14.3	10.0	0.4	5.3	0
Itching	24.2	9.1	10.4	0	23.3	0.4
Numbness and tingling	35.1	12.4	9.1	0.9	7.9	0
General pain (c)	42.4	20.4	16.5	4.3	13.9	0.8
Headache	19.1	4.3	2.6	0	4.9	0.4
Joint pain	38.5	22.1	4.8	0	9	0
Insomnia	41.1	22.1	9.5	0	6	0.4
Fatigue	53.3	23.4	29.4	2.6	25.9	3.8
Pain and swelling at injection site (b)	65.8	—	0.4	0	57.5	7.9

*Note:* Common Terminology Criteria for Adverse Events (CTCAE) was used by clinicians for symptom reporting and gradation. Patient‐Reported Outcomes Common Terminology Criteria for Adverse Events (PRO‐CTCAE) was used by patients for symptom reporting and gradation. Frequencies are reported in percentages (%). (a) Dry mouth was assessed in AMI trial in place of stomatitis symptom that was reported in the KEYNOTE 045 trial, but not assessable in the PRO‐CTCAE tool. (b) Symptoms “rash”, “diarrhea” and “Pain and swelling at injection site” did not have an evaluation of severity in the PRO‐CTCAE instrument. (c) General pain was assessed in AMI trial in place of back pain symptom that was reported in the KEYNOTE 045 trial, but not assessable in the PRO‐CTCAE tool. (d) Adverse event frequencies according to KEYNOTE 045 trial using CTCAE clinicians' assessment (Bellmunt et al. 2017).

Agreement score between clinicians and patients was assessable for 9 symptoms of the 17 eligible. A negative Cohen's kappa coefficient for symptom grade ≥ 3 of −0.017 (95% confidence interval (CI), [−0.039, 0.005]) for numbness and tingling symptom corresponded to bad agreement. The best kappa coefficient was 0.161 (95% CI, [0.045, 0.276]), for fatigue symptom, corresponding to poor agreement. Contingence chart and corresponding kappa agreement coefficient are summarized in Table 4.

**TABLE 4:** Contingence chart and kappa agreement scores for evaluable individual symptoms at baseline according to CTCAE or PRO‐CTCAE assessment.

CTCAE	PRO‐CTCAE		Kappa coefficient value (95% CI)	Concordance level
	Grade < 3 (%)	Grade ≥ 3 (%)		
Dry mouth				
Grade < 3 (%)	199 (86.15)	31 (13.42)	0.053 (−0.047, 0.153)	Poor
Grade ≥ 3 (%)	0 (0.00)	1 (0.43)		
Decrease appetite				
Grade < 3 (%)	208 (90.04)	20 (8.66)	0.140 (−0.046, 0.326)	Poor
Grade ≥ 3 (%)	1 (0.43)	2 (0.87)		
Nausea				
Grade < 3 (%)	220 (95.24)	10 (4.33)	−0.008 (−0.022, 0.006)	Bad
Grade ≥ 3 (%)	1 (0.43)	0 (0.00)		
Constipation				
Grade < 3 (%)	184 (79.66)	46 (19.91)	0.033 (−0.031, 0.098)	Poor
Grade ≥ 3 (%)	0 (0.00)	1 (0.43)		
Abdominal pain				
Grade < 3 (%)	204 (88.31)	25 (10.82)	−0.016 (−0.038, 0.005)	Bad
Grade ≥ 3 (%)	2 (0.87)	0 (0.00)		
Skin dryness				
Grade < 3 (%)	197 (85.28)	33 (14.29)	−0.008 (−0.025, 0.008)	Bad
Grade ≥ 3 (%)	1 (0.43)	0 (0.00)		
Numbness and tingling				
Grade < 3 (%)	197 (85.28)	32 (13.85)	−0.017 (−0.039, 0.005)	Bad
Grade ≥ 3 (%)	2 (0.87)	0 (0.00)		
General pain				
Grade < 3 (%)	179 (77.49)	42 (18.18)	0.112 (−0.014, 0.238)	Poor
Grade ≥ 3 (%)	5 (2.16)	5 (2.16)		
Fatigue				
Grade < 3 (%)	177 (76.62)	48 (20.78)	0.161 (0.045, 0.276)	Poor
Grade ≥ 3 (%)	0 (0.00)	6 (2.60)		

*Note:* Of the 17 analyzed symptoms only nine were eligible for *ĸ* coefficient estimation.

Abbreviations: 95% CI: 95% confidence interval; CTCAE: common terminology criteria for adverse events; PRO‐CTCAE: patient‐reported outcomes common terminology criteria for adverse events.

**FIGURE 3:**
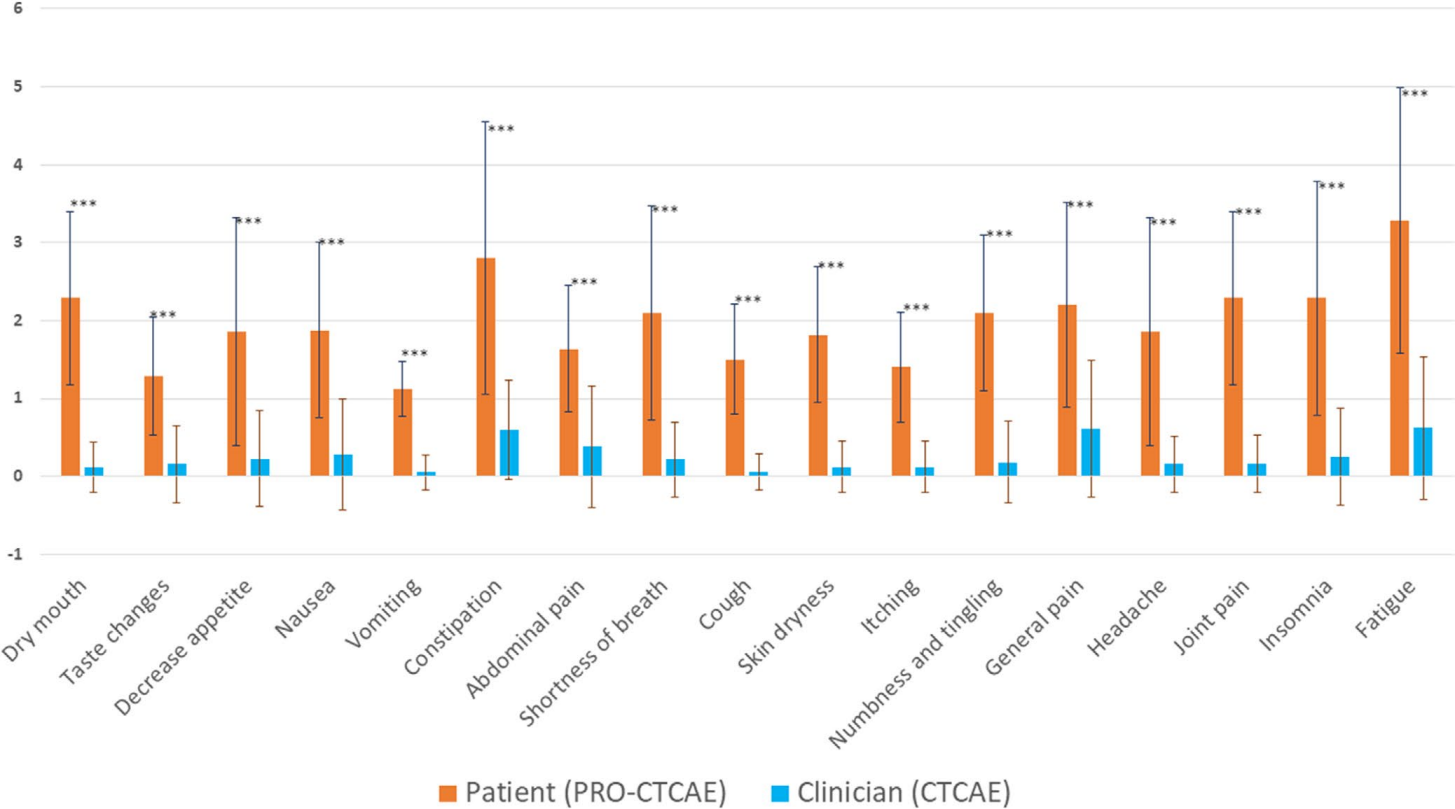
Toxicity index comparison for each symptom between clinician and patient assessment. Each bar corresponds to the mean symptom toxicity index. Confidence intervals correspond to standard deviations. * Symptoms “rash”, “diarrhea” and “Pain and swelling at injection site” did not have an evaluation of severity in the PRO‐CTCAE instrument. ***p value statistically significant (< 0.001). CTCAE: common terminology criteria for adverse events; PRO‐CTCAE: patients reported outcomes‐common terminology criteria for adverse events.

The comparison for toxicity index between clinicians and patients showed a statistically significant difference for all assessed symptoms (Figure 3). Wilcoxon test *p* values were all < 0.001 (Table S2).

## 4 Discussion

Here, we report an analysis of symptoms reported by patients under pembrolizumab treatment for la/mUC before treatment initiation until cycle 12. We compared the symptom assessment between clinicians and patients using validated tools and different statistical methods. This is the first report of the toxicity index comparison between clinician CTCAE and patient PRO‐CTCAE. PRO‐CTCAE questionnaires completion rate was stable across time, starting at 77.5% at inclusion and ending at 69.2% at cycle 12.

When compared to the KEYNOTE 045 trial (Table 3), clinicians under real–life conditions reported fewer symptoms in terms of frequency for most symptoms. Moreover, in the KEYNOTE 045 trial, grade ≥ 3 symptoms were more frequent than in our study. This was despite the fact that we had a list of 20 symptoms to rate, with a reminder of the severity scale. It could be explained by differences in the two cohorts. In our study, more patients were classed ECOG PS ≥ 2 (20% vs. 0.7%) and there were more older patients (median age 72.2 versus 67 years old) [1]. Another explanation could be that patients rarely report symptoms such as dry mouth or fatigue, as they may consider these symptoms to be normal.

According to the comparison of the frequencies of grade ≥ 3 symptoms, toxicity index and the Cohen's kappa coefficient, agreement between clinicians and patients for symptom severity was poor. Our results are in accordance with previously reported studies across other solid tumors in which clinicians underestimate symptom severity [29, 30]. At the individual level, using the toxicity index, we demonstrated a statistically significant discordance between clinicians and patients. The toxicity index is a robust way to assess AEs and to compare clinician and patient longitudinal reports [23, 25]. Its graphical representation is easy to understand by clinicians and easily compared between CTCAE and PRO‐CTCAE reports. This discrepancy may be due to clinicians and researchers focusing on measurable side effects and biological test results rather than subjective symptoms.

Our study is the first study assessing clinicians' versus patients' symptom severity reporting for la/mUC patients under an ICI, using two validated symptom recording tools. Our findings are in accordance with previous studies for patients treated with chemotherapy or other tumors under ICI [29, 30, 31, 32]. The discrepancy between clinicians and patients was also observed and maintained during follow‐up when using the toxicity index. The toxicity of pembrolizumab may be underestimated by clinicians in practice. Indeed, patients may be reluctant to report all side effects for fear of discontinuing treatment. Although pembrolizumab may be discontinued if it is poorly tolerated, particularly in patients with localized disease [33]. These patients may perceive discontinuation of the ICI less negatively than patients with advanced disease, as their prognosis is much better.

According to our findings, the PRO‐CTCAE instrument could be used in both routine practice and clinical research. First, its use in routine practice has been shown to improve HRQoL and survival in several real‐world studies, whereby patients completed the questionnaire electronically, with this information communicated back to their clinicians [10, 34, 35]. Electronic PRO monitoring during routine practice is helpful for medical decision and improves patient adherence to their therapeutic project according to PRO‐TECT trial results [34]. In phase III trials, the PRO‐CTCAE tool could help for symptom exhaustivity assessment as recommended by health society guidelines [9, 10, 36, 37, 38]. Moreover, it can be used in early phase I trials to determine dose‐limiting toxicity decision [38, 39, 40]. Indeed, for patients, it could contribute to the decision to improve the oncological treatment tolerance and to define the best recommended phase II dose.

However, our study had several limitations. First, symptoms selected for the PRO‐CTCAE questionnaire were based on the review of the most reported symptoms in the KEYNOTE 045 trial; thus, patients might have experienced other symptoms that were not included in the questionnaire [1]. Another limitation was the pre‐filled CTCAE questionnaire, which may have prompted physicians to pay close attention to all the pre‐specified symptoms. Therefore, clinicians might have reported some symptoms more frequently than in daily practice. However, the severity was likely to be reliable due to the recall strategy for CTCAE grading. Also, most symptoms were not graded ≥ 3 by clinicians, which limited the possibility of generating the Cohen's kappa coefficient. This is probably due to our symptom list, which consisted of less visible or sensitive symptoms and the small sample size. Moreover, diarrhea severity was highly reported by clinicians; however, it was not assessable for severity in the PRO‐CTCAE, but only for frequency. A correlation between frequency and severity could be made in the same way as for the CTCAE severity definition. Nevertheless, we preferred to stick strictly to the PRO‐CTCAE item definitions.

## 5 Conclusions

To our knowledge, this is the first prospective real‐life study specifically assessing patient symptoms using the PRO‐CTCAE for la/mUC patients under pembrolizumab. Our blinded comparative severity assessment showed a poor agreement between clinicians and patients. These results were similar using three different statistical methods. Moreover, the longitudinal symptom assessment showed significant differences between clinicians and patients. Clinicians reported fewer symptoms than patients and rated them as less severe than patients. PRO‐CTCAE measurement should be used in early and late clinical trials and implemented in routine practice to help patient management by early and better detection of subjective symptoms.

## Author Contributions

Conceptualization: S.A., F.F., N.H. Data curation: S.A., F.F., N.H. Formal analysis: E.D.M., F.F. Funding acquisition: S.A., N.H. Investigation: S.A., G.R., P.F., A.G., D.B., D.P., E.B., R.D., P.B., S.O., C.T., D.T., N.H. Methodology: S.A., T.C., F.F., N.H. Supervision: N.H. Validation: All authors. Roles/writing – original draft: S.A. Writing – review and editing: All authors.

## Ethics Statement

The study was conducted in accordance with the Declaration of Helsinki, and the protocol was approved by a French ethical committee (*Comité de protection des personnes Ile‐de‐France IV*, #2020/56).

## Consent

All patients provided written informed consent to participate.

## Conflicts of Interest

D.B.: Astellas, Gilead, Janssen, Merck, MSD, Pfizer, Seagen. C.T.: AAA, Amgen, AstraZeneca, Astellas, BMS, Ipsen, Janssen, Merck, MSD, Pfizer, Sanofi. P.B.: MSD. N.H.: Astellas, AstraZeneca, Bayer, BMS, Ipsen, Janssen, Merck, Pfizer. The authors declare no conflicts of interest.

## Supporting information


**Table S1.** Wilcoxon test results for grade ≥ 3.
**Table S2.** Toxicity index for each symptom.

## Data Availability

The data that support the findings of this study are available from the corresponding author upon reasonable request.
